# Suppressive Effect of Coffee Leaves on Lipid Digestion and Absorption In Vitro

**DOI:** 10.3390/foods13152445

**Published:** 2024-08-02

**Authors:** Veerawat Sansri, Morakot Sroyraya, Pochamana Phisalprapa, Atchariya Yosboonruang, Atcharaporn Ontawong, Surasak Saokaew, Bey-Hing Goh, Kanittaporn Trisat, Premchirakorn Phewchan, Anchalee Rawangkan, Nanteetip Limpeanchob, Acharaporn Duangjai

**Affiliations:** 1 Department of Basic Medical Science, Faculty of Medicine Vajira Hospital, Navamindradhiraj University, Bangkok 10300, Thailand; veerawat@nmu.ac.th; 2 Department of Anatomy, Faculty of Science, Mahidol University, Bangkok 10400, Thailand; 3 Division of Ambulatory Medicine, Department of Medicine, Faculty of Medicine Siriraj Hospital, Mahidol University, Bangkok 10700, Thailand; 4 Division of Microbiology, School of Medical Sciences, University of Phayao, Phayao 56000, Thailand; 5 Unit of Excellence in Research and Product Development of Coffee, Division of Physiology, School of Medical Sciences, University of Phayao, Phayao 56000, Thailand; 6 Center of Health Outcomes Research and Therapeutic Safety (Cohorts), School of Pharmaceutical Sciences, University of Phayao, Phayao 56000, Thailand; 7 Unit of Excellence on Clinical Outcomes Research and Integration (UNICORN), School of Pharmaceutical Sciences, University of Phayao, Phayao 56000, Thailand; 8 Unit of Excellence on Herbal Medicine, School of Pharmaceutical Sciences, University of Phayao, Phayao 56000, Thailand; 9 Division of Social and Administrative Pharmacy, Department of Pharmaceutical Care, School of Pharmaceutical Sciences, University of Phayao, Phayao 56000, Thailand; 10 Biofunctional Molecule Exploratory Research Group (BMEX), School of Pharmacy, Monash University Malaysia, Bandar Sunway 47500, Malaysia; 11 College of Pharmaceutical Sciences, Zhejiang University, Hangzhou 310058, China; 12 Department of Pharmacy Practice and Center of Excellence for Innovation in Chemistry, Pharmacological Research Unit, Faculty of Pharmaceutical Sciences, Naresuan University, Phitsanulok 65000, Thailand

**Keywords:** coffee leaves, pancreatic lipase, cholesterol micelles, bile acid binding, lipid absorption

## Abstract

Background: Coffee leaves are a major source of bioactive components and are used as ethnomedicine. However, despite their traditional medicinal use, information about their effects on antihyperlipidemia remains limited. Methods: The aims of this study were to evaluate the main components of leaf extracts from Arabica and Robusta coffees and to examine the potential of these coffee leaves in reducing lipid digestion and absorption in vitro. Results: Coffee leaf extracts from Arabica coffee contain a high amount of caffeine, whereas extracts from Robusta coffee contain high amounts of chlorogenic acid (CGA) and caffeine. Additionally, leaf extracts from Arabica and Robusta coffee demonstrated the inhibition of pancreatic lipase, decreased micellar cholesterol solubility, and reduced bile acid binding. Furthermore, these extracts resulted in a reduction in cholesterol uptake in Caco-2 cells. Molecular docking experiments supported this discovery, showing CGA and caffeine binding to Niemann–Pick C1-like 1 (NPC1L1), a key protein in cholesterol absorption. The results indicated that CGA and caffeine can competitively bind to NPC1L1 at the cholesterol binding pocket, reducing its cholesterol binding rate. These findings suggest that coffee leaves might help suppress lipid absorption and digestion, highlighting their potential use in preventing and treating hyperlipidemia.

## 1. Introduction

High levels of plasma cholesterol are a significant risk factor for atherosclerosis and coronary heart diseases. Plasma cholesterol levels are influenced by a variety of factors, including absorption in the gut, de novo cholesterol synthesis, and cholesterol removal from the bloodstream [1]. Recently, an additional important role of the intestine in the maintenance of cholesterol homeostasis and regulation of plasma cholesterol levels has become apparent [2]. The intestine plays a significant role in cholesterol regulation, and it appears that lowering LDL cholesterol levels can be achieved by the inhibition of cholesterol absorption, maintaining it within a low-normal range [3].

Coffee leaves have been used traditionally in India, Jamaica, and Java for various purposes, such as promoting lactobacillus growth, serving as organic fungicides, improving animal feed, and as ethnomedicine to enhance health [4]. Moreover, coffee leaves have been utilized for medicinal purposes across various regions of the world. In Haiti, they are used to treat anemia, edema, and asthenia, while in Africa, they are used to alleviate diarrhea and intestinal pain. In Uganda, coffee leaves are used to provide relief from HIV/AIDS-related pain, and in Cuba and Nicaragua, they are used to relieve migraine pain. In Peru, they are utilized to treat cough associated with flu and lung conditions, and in Mexico, they are used to treat fever [5].

Coffee leaves contain a wide range of bioactive compounds, including alkaloids (such as caffeine, adenine-7-glucosyl, trigonelline, theobromine, and 7-methylxanthine), flavonoids (like anthocyanins, quercetin, isoquercitrin, quercetin glucoside, rutin, and kaempferol), terpenoids (such as cafestol, kahweol, and 16-O-methyl cafestol), amino acids (histidine and pipecolic acid), sucrose, tannins, xanthonoids (isomangiferin and mangiferin), phenolic acids (chlorogenic, caffeic, sinapic, p-coumaric, ferulic acids, 3-caffeoyl-quinic acid, 5-caffeoyl-quinic acid, 4-caffeoyl-quinic acid, 4-feruloyl-quinic acid, 5-feruloyl-quinic acid, 3,4-dicaffeoyl-quinic acid, 3,5-dicaffeoyl-quinic acid, 4,5-dicaffeoyl-quinic acid), and catechins (including epicatechin and catechin) [5,6,7,8,9]. Previous research findings demonstrated that administering coffee leaf extracts led to decreased blood glucose, total cholesterol, total triglycerides, and LDL levels, while also increasing HDL levels [10]. Moreover, the study revealed the anti-obesity potential of coffee leaf extract by reducing hepatic lipid accumulation, decreasing adipocyte size in adipose tissue, and downregulating the expression of lipogenic transcription factors [10]. Additionally, the extracts were found to reduce lipid peroxidation in both the plasma and aorta in in vivo studies [11]. Despite extensive research on coffee bean and coffee pulp extracts concerning lipid digestion and absorption [12,13], the effects of coffee leaves have largely remained unexplored. This gap in knowledge is significant, particularly because the main bioactive components, such as chlorogenic acids (CGAs) and caffeine, vary in proportion between coffee leaves and other parts of the coffee plant [4,14,15]. Understanding the unique profile and potential health benefits of coffee leaves could open new avenues for nutritional science and health applications. Thus, the objective of this study was to investigate the potential of coffee leaf extract from Arabica and Robusta coffee on lipid digestion and absorption. Specifically, we aimed to assess its impact on reducing pancreatic lipase activity, micellar cholesterol solubility, bile acid binding, and cholesterol uptake in Caco-2 cells. Additionally, we sought to explore the mechanisms through which coffee leaf extracts may reduce cholesterol uptake in this intestinal epithelial cell line.

## 2. Materials and Methods

### 2.1. Materials and Reagents

All other chemicals utilized in this research were of analytical grade. The pancreatic lipase (type II, sourced from porcine pancreas), 1, 2 di-O-lauryl-rac-glycero-3 glutaric acid 6-methyl resorufin ester, orlistat, cholesterol, l-α-phosphatidylcholine, taurocholic acid sodium salt hydrate, taurodeoxycholic acid, glycodeoxycholic acid, hydrazine hydrate solution, 3-α-hydroxysteroid dehydrogenase, β-nicotinamide adenine dinucleotide phosphate hydrate (NAD), and cholestyramine were purchased from Sigma-Aldrich Co. (located in St. Louis, MO, USA). Cholesterol test kit was purchased from HUMAN GmbH Co. (Wiesbaden, Germany). Dulbecco’s modified Eagle’s medium (DMEM/F12), fetal bovine serum, trypsin–EDTA, and penicillin–streptomycin were obtained from Gibco (Grand Island, NY, USA). The 23-(dipyrrometheneboron difluoride)-24-norcholesterol (TopFluor^®^ Cholesterol) was purchased from Avanti Polar Lipids, Inc. (Alabaster, AL, USA). Additionally, 3-(4,5- dimethylthiazol-2-yl)-2,5-diphenyltetrazolium bromide (MTT); dimethyl sulfoxide (DMSO); ethanol; ezetimibe; and potassium phosphate buffer were used.

### 2.2. Plant Material

The leaves of Arabica coffee (*Coffea arabica*) and Robusta coffee (*Coffea robusta*) were collected at Ban Khun Kong, Wiang Haeng district, Chiang Mai province, northern Thailand, and provided by the Chao-Thai-Pukao Factory, Chiang Mai, Thailand. The leaves were extracted with hot water (1:5; *w*/*v*) for 10 min. The filtered solutions were freeze dried in a freeze drier after three repeats of this operation. The crude extracts of Arabica leaf and Robusta leaf were kept at –20 °C until further analysis.

### 2.3. Determination of Proximate Composition

The proximate composition analysis of moisture, dry matter, fiber, ash, fat, protein, nitrogen-free extract (NFE), and energy content in the leaf samples from Arabica and Robusta was conducted following the AOAC protocol methods [16].

### 2.4. Determination of High-Performance Liquid Chromatography (HPLC) Analysis

The quantities of caffeine and chlorogenic acid (CGA) in coffee leaf extracts were determined using HPLC. The HPLC analysis was conducted using an Agilent 1200 series HPLC instrument (Shimadzu, Kyoto, Japan) equipped with a UV detector set at 320 nm for CGA and caffeic acid and at 280 nm for caffeine. The chromatographic separation was conducted using an Inertsil ODS-3-C18 column (4.6 × 150 mm, particle size 5 µm, GL Sciences Inc., Shinjuku-ku, Tokyo, Japan) with an injection volume of 20 µL and a flow rate of 0.8 mL/min. The mobile phase consisted of solvent A (2% (*w*/*v*) acetic acid) and solvent B (methanol). The gradient was programmed as follows: 12% B from 0 to 9 min; 12–15% B from 9 to 14 min; 15–30% B from 14 to 24 min; 30–95% B from 24 to 28 min; 95–99% B from 28 to 36 min; 95–99% B from 36 to 41 min; and 99.5–12% B from 41 to 42 min. The chromatographic peaks were distinguished by comparing their retention times to established standards. The concentration of each component was then measured by calculating the peak area under the curve relative to a standard.

### 2.5. Determination of Lipid Digestion and Absorption In Vitro

#### 2.5.1. Determination of Pancreatic Lipase Inhibition

Pancreatic lipase activity was measured using the method of Aubry et al. [17]. Extracts were prepared at varying concentrations with reaction buffer at pH 8.0 (0.8 M Tris-HCl, 150 mM NaCl, and 1.3 mM CaCl_2_). A volume of 25 µL of extract was mixed with 40 µL of reaction buffer and 25 µL of 50 U/mL pancreatic lipase in a 96-well plate (black side and clear bottom). Finally, the reaction was started by adding 10 µL of 400 µM substrate (1,2-di-O-lauryl-rac-glycero-3-glutaric acid 6′-methylresorufin ester) and run at 37 °C in the dark for 60 min. The amount of methylresorufin was measured as fluorescence (Ex 535 nm and Em 595 nm). Orlistat (tetrahydrolipstatin), a drug used to treat obesity, was employed as a positive control due to its established efficacy as a pancreatic lipase inhibitor. It binds to the active sites of pancreatic lipases, thereby inhibiting the hydrolysis of dietary fats into absorbable free fatty acids [18,19]. Pancreatic lipase inhibition was calculated using the following formula:

$$\%\;\mathrm{Pancreatic}\;\mathrm{lipase}\;\mathrm{inhibition}\;=\;\frac{{[\mathrm{Absorbance}}_{\left(\mathrm{control}\right)}-{\mathrm{Absorbance}}_{\left(\mathrm{sample}\right)}]}{{\mathrm{Absorbance}}_{\left(\mathrm{control}\right)}}\;\times 100$$

#### 2.5.2. Determination of Cholesterol Micelles’ Solubility

Cholesterol micelle solutions (comprising 0.6 mM phosphatidylcholine, 1 mM cholesterol, and 1 mM sodium taurocholate) were prepared following the method of Kirana et al. (2005) [20], with slight modifications. The leaf extracts or cholestyramine (100–2000 µg/mL) were incubated with the micelle solutions for a duration of 3 h. Cholestyramine, a well-known cholesterol-reducing agent, was used as the positive control. The concentration of cholesterol in the filtrate was subsequently determined as cholesterol micellar solubility using cholesterol assay kits.

#### 2.5.3. Determination of Bile Acid Binding

As mentioned in the previous work by Yoshie-Stark and Wäsche (2004) [21], the bile acid binding assay underwent slight modifications. In this experiment, taurocholic acid (TC), glycodeoxycholic acid (GC), and taurodeoxycholic acid (TD) were utilized as bile acids, while cholestyramine served as the positive control. In summary, 200 mL of coffee leaf extracts or cholestyramine (100–2000 µg/mL) were incubated with 200 mL of bile acid (2 mM) in 100 mM PBS at pH 7.0 for 2 h at 37 °C. To separate the bound bile acids from the free ones, the mixed solutions were subjected to centrifugation at 10,000 rpm for 10 min and subsequently passed through a 0.22 µm membrane. The bile acid concentration was determined following the total bile acids procedures as shown below. The reaction mixtures containing 0.133 mol/L tris buffer (pH 9.5), 1 mol/L hydrazine hydrate, and 7.7 mmol/L nicotinamide adenine dinucleotide (NAD) were combined with the filtrated bile acid solutions. Additionally, 10 L of 1 unit/mL 3-hydroxysteroid dehydrogenase were added to the mixture. The combined solutions were then incubated for 90 min at 30 °C. The production rate of thio-nicotinamide adenine dinucleotide (NADH) was assessed by measuring specific changes in absorbance at 405 nm using a microplate reader.

### 2.6. Cholesterol Uptake in Caco-2 Cells

#### 2.6.1. Determination of Cell Viability

Caco-2, a colon carcinoma cell line, was acquired from the American Type Culture Collection (ATCC). These cells were cultured in DMEM/F12 supplemented with 10% fetal bovine serum and 1% penicillin–streptomycin at 37 °C in a humidified environment with 95% humidity. Trypsin–EDTA was used to suspend the cells, which were then plated in 96-well plates or culture flasks. The MTT assay was employed to determine cell viability. In 96-well plates, cells were plated at a density of 10,000 cells per well and incubated for 24 h. After 22 h of incubation with coffee leaf extracts (100–1000 µg/mL), 10 µL of MTT (5 mg/mL) was added, and the solution was incubated for another 2 h. The culture medium was then withdrawn, and the formazan dye was solubilized using 200 µL of dimethyl sulfoxide:ethanol (1:1) before measuring the absorbance at 595 nm. The data are represented as the percentage of viable cells.

#### 2.6.2. Determination of Cholesterol Uptake in Caco-2 Cells

The uptake experiment was performed using a micellar cholesterol solution. Briefly, the micellar solution had the following final concentrations: 0.1 mM NBD-cholesterol, 1 µM cholesterol, 50 µM phosphatidylcholine, and 2 mM sodium taurocholate. After cell differentiation, Caco-2 cells were subjected to a 1 h pre-treatment with coffee leaf extracts (1000 µg/mL) or a positive control, ezetimibe (400 µM). Subsequently, a cholesterol micelle solution was added and incubated for 3 h. Following incubation, the fluorescent intensity was measured at an excitation wavelength of 485 nm and an emission wavelength of 535 nm. The cells were then washed twice with ice-cold PBS before being lysed using 0.2 N NaOH/0.1% Triton-X 100. The protein content of the cellular lysates was determined through a BCA colorimetric assay.

### 2.7. Molecular Docking

The structure of CGA and caffeine, which are the primary active components in extracts from Arabica and Robusta coffee leaves, was sourced from https://pubchem.ncbi.nlm.nih.gov (accessed on 4 December 2023). The crystal structure of the N-terminal domain of Niemann–Pick C1-like 1 (NPC1L1) (PDB code 3QNT) was obtained from the RCSB Protein Data Bank. Due to the presence of a distinct cholesterol-binding site within the structure of NPC1L1′s N-terminal domain (NTD), the NPC1L1 (NTD) structure was suitable for conducting docking experiments. The NPC1L1 (NTD) data were accessed using AutoDock Tools (version 1.5.6), and the N-terminal domain’s structure was preserved as a PDBQT receptor file. Utilizing AutoDock Tools, the number of points in dimensions (X = 52, Y = 72, Z = 66) and center grid box (X = −19.244, Y = −25.055, Z = −19.052) were determined on a 0.375 angstrom scale for the N-terminal domain. The number of points in dimensions, center point, and spacing were all detailed in a configuration file. The configuration file created was used to run AutoDock Vina [22]. The binding affinity of each ligand was obtained from AutoDock Vina by utilizing a configuration file for input data. The dimensions and positions of the binding sites were displayed using PyMOL 2.5.5.

### 2.8. Statistical Analyses

The data were provided as a mean with a standard error of the mean (SEM). Statistical significance was determined using paired two-tailed Student’s *t*-tests, with a significance level of *p* ≤ 0.05 considered statistically significant.

## 3. Results and Discussion

### 3.1. Nutrition Components and Bioactive Compounds in Coffee Leaf Extract

The nutritional composition of Arabica and Robusta coffee leaves was analyzed to determine their moisture, dry matter, fiber, ash, fat, protein, NFE (representing nonfibrous carbohydrates, such as sugars and starches), and energy content. As presented in Table 1, there were no significant differences in the proximate composition between the leaves of Arabica and Robusta, except for ash, protein, and NFE, where the differences were statistically significant (*p* < 0.05). Arabica coffee leaves displayed lower ash content (5.59%) compared to Robusta (6.99%), possibly indicating higher mineral content in Robusta. Arabica exhibited higher protein content (17.73%) compared to Robusta (14.33%), suggesting greater protein abundance. Additionally, Arabica had a slightly higher %NFE (18.03%) than Robusta (16.15%), indicating a slightly greater proportion of soluble carbohydrates.

**Figure 1:**
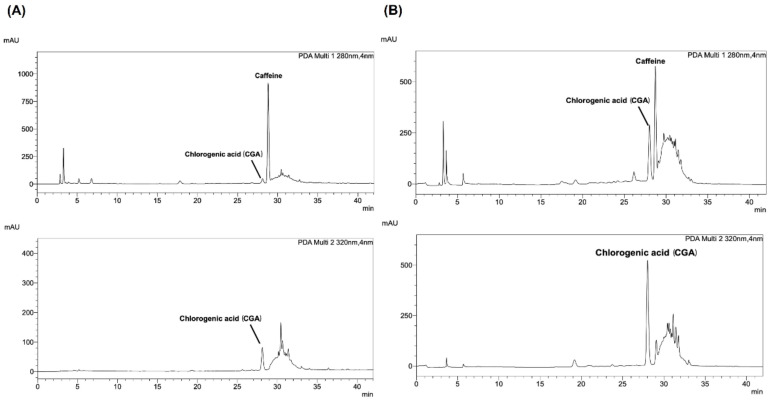
The HPLC chromatograms of (**A**) Arabica and (**B**) Robusta coffee leaf extracts.

The coffee leaf extracts were then subjected to HPLC analysis to identify their major compounds. Additionally, the quantities of caffeine and CGA in the extracts were determined using HPLC. Figure 1 presents the chromatographic profiles of Arabica and Robusta coffee leaf extracts. The UV wavelength for the detection of the two components was UV 320 nm for CGA and UV 280 nm for caffeine. The amount of CF and CGA was quantified with a calibration curve of the peak area of standard caffeine and CGA in the concentration range of 0.78 to 100 µg/mL with limits of detection (LODs) found to be 0.0125 and 0.05 μg/mL and limits of quantitation (LOQs) 0.05 and 0.10 μg/mL, respectively. The retention time periods for CGA and caffeine were found to be 27.997–27.970 min and 27.989–28.679 min, respectively. The calibration curves for CGA and caffeine were y = 24304x + 7895.2 (R^2^ = 0.9994) and y = 55777x − 13020 (R^2^ = 0.9999), respectively. Arabica extracts exhibited significantly lower levels of CGA (2.91 ± 0.22 mg/g extract) compared to Robusta (18.15 ± 0.91 mg/g extract). Conversely, the Arabica extracts contained higher concentrations of caffeine (19.82 ± 0.75 mg/g extract) compared to Robusta (14.90 ± 0.76 mg/g extract), as shown in Table 2. These findings suggest distinct chemical profiles between Arabica and Robusta coffee leaves.

According to previous reports, the primary antioxidants present in coffee are chlorogenic acids (CGAs), which are esters derived from caffeic acid and quinic acid [23,24,25,26]. CGA and its related compounds are considered to exhibit a diverse array of beneficial effects across various biological domains. These effects encompass antioxidant activity, antidiabetic, DNA protective, and neuroprotective activities [23,24,27,28]. Furthermore, the CGAs have shown protective effects against in vitro LDL oxidation [25,29,30]. A previous in vivo study also found that CGA reduces plasma total cholesterol, LDL, and hepatic lipid levels while increasing HDL, leading to improved atherogenic indices and cardiac risk factors [31].

### 3.2. Arabica and Robusta Coffee Leaf Extracts Exhibited Inhibitory Effects on Lipase Activity

**Figure 2:**
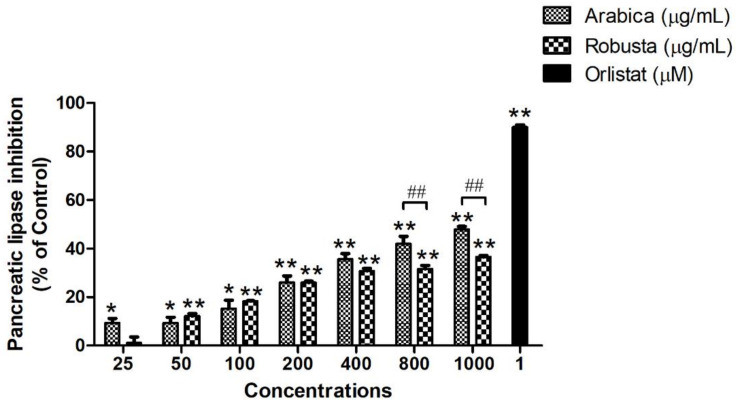
Pancreatic lipase inhibitory activity of Arabica and Robusta coffee leaf extracts. Data are represented as a percentage of the control. The data represent the mean ± SEM (*n* = 3); ** *p* < 0.001; * *p* < 0.05 compared with the control; ## *p* < 0.001 compared between Arabica and Robusta coffee leaf extracts.

The lipase inhibitory effects of the Arabica and Robusta coffee leaf extracts are indicated as a percentage (%). Figure 2 illustrates the dose–response inhibition of lipase activity by the Arabica and Robusta coffee leaf extracts. Orlistat, known for its lipase inhibitory properties, shows a nearly 100% inhibition, serving as a positive control to assess the effectiveness of the coffee leaf extracts. Both the Arabica and Robusta extracts are effective in inhibiting pancreatic lipase activity, with the degree of inhibition increasing in a concentration-dependent manner. Notably, the Arabica extracts consistently demonstrate higher inhibition across most concentrations compared to the Robusta extracts, particularly at 800 and 1000 µg/mL (*p* < 0.001). These findings suggest that coffee leaf extracts, especially from Arabica, have significant potential as natural inhibitors of pancreatic lipase.

Pancreatic lipase is a crucial enzyme involved in the digestion and absorption of dietary fats. Decreasing fat absorption by inhibiting pancreatic lipase is recognized for its beneficial impact on managing obesity. A known pancreatic lipase inhibitor, 1 µM orlistat, was employed as a reference compound [32]. Our findings align with prior observations indicating that GCA has a suppressing effect on pancreatic lipase activity, as documented in earlier studies by Yuniarti et al. (2019) [33] and Hu et al. (2015) [34]. The research revealed that CGA acted as a competitive inhibitor of porcine pancreatic lipase, binding noncovalently to the location housing its catalytic triad. This interaction occurred with the catalytic triad components in pancreatic lipase, specifically Ser153, His264, and Asp177, as elucidated in the work by Hu et al. (2015) [34]. In the meantime, caffeine exhibited the capacity to attach itself to both free enzymes and an enzyme–substrate complex. Caffeine significantly inhibited the process of lipid hydrolysis, which is facilitated by the pancreatic enzyme. Consequently, when caffeine binds to free enzymes, it impedes the interaction between the enzyme and the substrate, thereby hindering the formation of the final product. Moreover, caffeine exhibited a higher effectiveness in inhibiting the lipolysis of short-chain fatty acid substrates compared to longer-chain substrates [35].

### 3.3. The Effect of Arabica and Robusta Coffee Leaf Extracts on the Micellar Solubility of Cholesterol and Bile Acid Binding

The effects of the Arabica and Robusta coffee leaf extracts on the micellar solubility of cholesterol and bile acid binding are presented in Table 3. Cholestyramine, a drug that binds bile acids to prevent their reabsorption in the digestive system and lower cholesterol levels, was utilized as the positive control. The data indicate that both Arabica and Robusta coffee leaf extracts have the potential to inhibit cholesterol micellar solubility and bile acid binding, albeit with varying efficacy depending on the specific bile acid. The Arabica and Robusta coffee leaf extracts exhibit comparable IC_50_ values for inhibiting cholesterol micellar solubility, with values of 2527 ± 1.09 µg/mL and 2542 ± 1.18 µg/mL, respectively. These IC_50_ values indicate that both Arabica and Robusta extracts have similar efficacy in this aspect. However, both types of coffee leaf extracts are significantly less effective than cholestyramine, which has a much lower IC_50_ value.

The micellar solubility enables the transport of lipid digestion products to the small intestinal surface for absorption. Consequently, numerous studies have identified a novel therapeutic target for managing hyperlipidemia and obesity by attenuating cholesterol absorption through the restriction of cholesterol micellization within the intestinal lumen [20]. Our results indicate that, while both the Arabica and Robusta coffee leaf extracts demonstrate potential in inhibiting cholesterol micellar solubility, their efficacy is surpassed by cholestyramine, suggesting the need for further exploration into their mechanisms and optimization for enhanced effectiveness in mitigating cholesterol absorption.

The bile acid binding experiment revealed that the Arabica leaf extracts exhibit a lower IC_50_ for TC compared to the Robusta extracts, indicating the superior binding efficiency of the Arabica extracts. For TD, both Arabica and Robusta exhibit similar IC_50_ values, which are significantly higher than those of cholestyramine. For GC, the Robusta extracts exhibit a slightly lower IC_50_ compared to the Arabica extracts, indicating the better binding efficiency of the Robusta extracts in this case. In summary, the Arabica extracts were more effective in binding TC, whereas the Robusta extracts show better binding efficiency for GC.

A promising approach to reducing plasma cholesterol levels involves binding bile acids and enhancing their fecal excretion. Cholestyramine, a bile acid sequestrant, plays a key role in this mechanism by sequestering bile acids and preventing their reabsorption from the gut, thereby disrupting the enterohepatic circulation of bile acids. As a consequence, the bile acid pool is reduced, leading to the increased conversion of cholesterol into bile acids and ultimately resulting in lowered plasma cholesterol levels [36]. Evidence has shown that CGA and caffeine have the potential to act on lipids by being able to bind to bile acids in vitro. This suggests that they may also reduce the functionality of bile acids in lipid digestion [12].

### 3.4. Arabica and Robusta Coffee Leaf Extracts Inhibited Cholesterol Uptake in Caco-2 Cells

**Figure 3:**
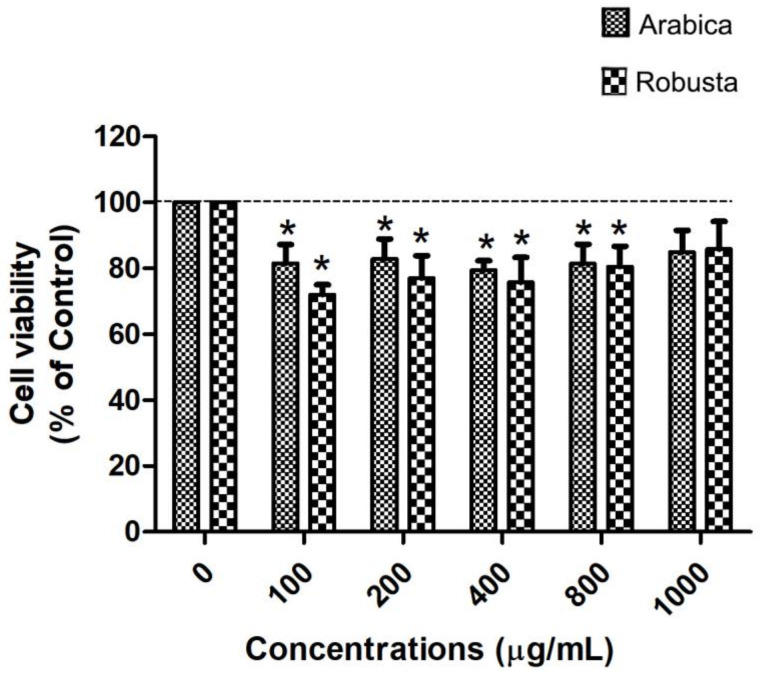
Cell viability of Caco-2 cells treated with Arabica or Robusta coffee leaves’ extract at 100–1000 µg/mL for 24 h; * *p* < 0.05 compared with control (*n* = 3).

The human Caco-2 cell line, which serves as a well-established in vitro model of the intestinal epithelial barrier, was utilized to investigate cholesterol uptake into the cells. The initial step involved conducting a cytotoxicity test on Arabica and Robusta coffee leaf extracts in Caco-2 cells. The results indicated that treatment with the Robusta coffee leaf extract resulted in Caco-2 cell viability of >80%, whereas treatment with the Arabica coffee leaf extract resulted in a cell viability of >75%. Additionally, at the concentration of 1000 μg/mL, no cytotoxic effects were observed on Caco-2 cells (Figure 3). These findings suggest that the tested concentrations of the compounds were non-toxic to the cells. Therefore, this specific concentration was selected for further investigations.

**Figure 4:**
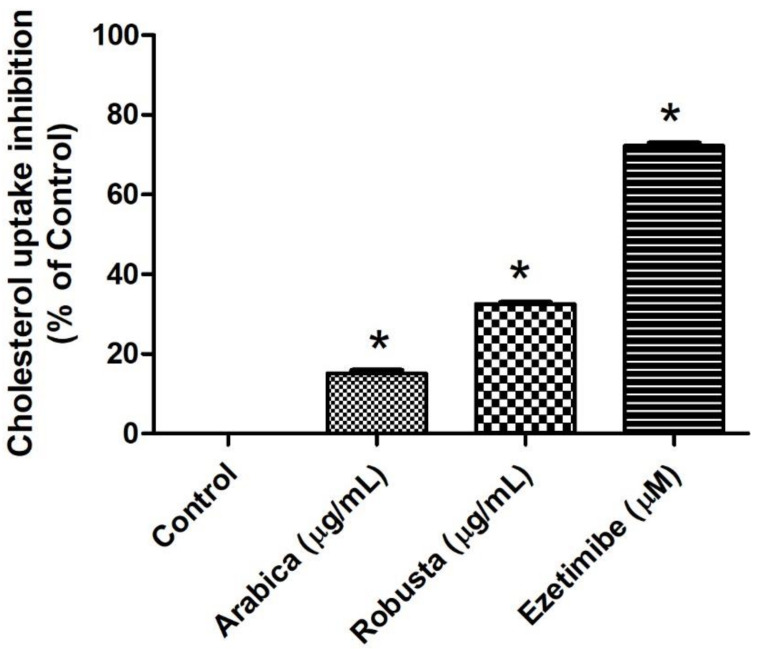
Cholesterol uptake inhibition in differentiated Caco-2 cells treated with Arabica or Robusta coffee leaves (1000 µg/mL) or ezetimibe (400 µM); * *p* < 0.05 compared with control (*n* = 3).

Afterwards, the potential of extracts from Arabica and Robusta coffee leaves to inhibit cholesterol absorption was investigated. Caco-2 cells were pre-treated for 24 h with either the Arabica or Robusta coffee leaf extracts (at a concentration of 1000 µg/mL) or we utilized ezetimibe (at 400 µM) as a positive control [37]. The results indicated that both the Arabica and Robusta coffee leaf extracts significantly inhibited cholesterol uptake compared to the control group. Specifically, the Arabica coffee leaf extract resulted in a 15% reduction of cholesterol uptake, while the Robusta coffee leaf extract achieved a 32% reduction (Figure 4). However, both coffee leaf extracts were considerably less effective than Ezetimibe, the positive control, which demonstrated a 72% reduction of cholesterol uptake.

### 3.5. CGA and Caffeine Can Bind to Niemann–Pick C1-Like 1 (NPC1L1) at the Cholesterol Binding Pocket

**Figure 5:**
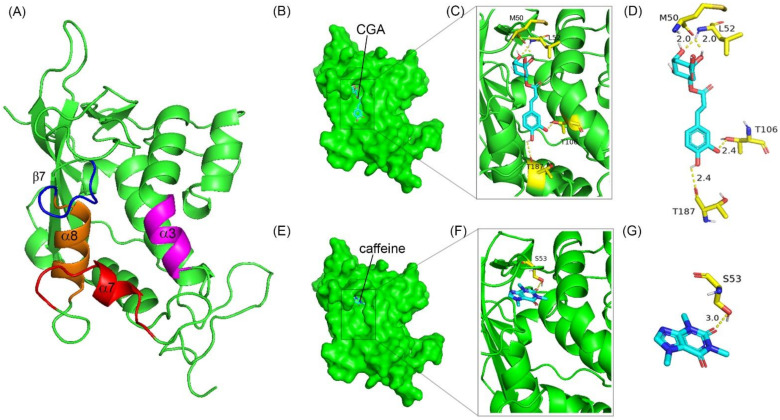
(**A**) A ribbon diagram illustrates the structure of NPC1L1-NTD (colored green). The α-helices and β-sheets encircling the entrance to the cholesterol binding pocket are represented using the colors magenta (α3), red (α7), orange (α8), and blue (β7). (**B**,**E**) show the surface representation of NPC1L1-NTD and the cholesterol-binding site with chlorogenic acid (CGA) and caffeine, respectively, shown in cyan sticks. (**C**,**F**) display ribbon diagrams depicting the interaction of CGA and caffeine with NPC1L1-NTD (PDB code 3QNT), respectively. Essential residues involved in CGA and caffeine binding are represented as yellow sticks, while CGA and caffeine are visualized as cyan sticks. Hydrogen bonds are illustrated using yellow dashed lines. (**D**,**G**) depict detailed interactions of the NPC1L1-NTD cholesterol-binding cavity with CGA and caffeine, respectively. Yellow sticks represent the crucial residues involved in ligand binding, while cyan sticks depict the ligands themselves. Yellow dashed lines are used to depict hydrogen bonds, and the distances of these hydrogen bonds are measured in angstroms (Å).

To elucidate whether CGA and caffeine, the primary active compounds in Arabica and Robusta coffee leaf extracts, can competitively bind at the cholesterol-binding site of the NPC1L1, we proceeded to conduct molecular docking. This involved assessing the binding affinity of CGA and caffeine with the cholesterol-binding site located within the N-terminal domain of NPC1L1 (NPC1L1-NTD) [38,39]. The results of the docking analysis can be observed in Figure 5 and Table 4. The findings indicate that both CGA and caffeine possess the capability to bind to the cholesterol-binding site of NPC1L1-NTD. The interaction between NPC1L1-NTD and CGA involves four specific amino acid residues: MET 50, LEU52, THR106, and THR187. In contrast, caffeine only interacts with a single amino acid residue, SER 53.

The interaction of CGA and caffeine with the critical amino acids in the NPC1L1-NTD suggests that both CGA and caffeine can effectively inhibit cholesterol absorption. When considering the inhibition of cholesterol binding uptake (as shown in Figure 4), it becomes evident that the Robusta coffee leaf extract, which contains a higher concentration of CGA and demonstrates greater binding affinity, exerts a more pronounced effect on inhibiting cholesterol binding uptake compared to the Arabica coffee leaf extract, which contains a higher proportion of caffeine than CGA. This suggests that the presence and concentration of CGA in coffee leaf extracts are crucial determinants of their efficacy in inhibiting cholesterol uptake.

This study provides clear evidence that Arabica and Robusta coffee leaf extracts exert inhibitory effects on cholesterol uptake by intestinal epithelial cell lines. The potential mechanism underlying this effect may be associated with NPC1L1, a transmembrane protein known to facilitate intestinal cholesterol uptake [12,40,41]. NPC1L1 is predominantly enriched in the apical membrane of the small intestine, where it plays a crucial role in the absorption process by mediating extracellular sterol transport across the brush border membrane [42]. Furthermore, earlier research indicated that the inhibition of NPC1L1 is associated not only with a decrease in cholesterol absorption but also with lower plasma cholesterol levels [12,41,43]. However, the effect of Arabica and Robusta coffee leaf extracts on lowering plasma cholesterol levels requires further investigation. Comprehensive studies, including randomized, controlled trials and mechanistic research, are needed to elucidate their bioactive compounds, optimal dosages, and long-term safety profiles. Unlocking the full potential of these natural extracts could pave the way for innovative and effective treatments for managing cholesterol and promoting cardiovascular health.

Despite the effectiveness of coffee extracts, including those from beans, pulp, and leaves, in reducing cholesterol levels, regular coffee consumption does not lead to lower blood cholesterol levels compared to non-consumption [44,45,46]. Furthermore, certain studies have observed that individuals who consume coffee without adding milk and sugar tend to exhibit higher cholesterol levels compared to non-coffee drinkers [47,48,49]. This association may be attributed to diterpenes in coffee oil, specifically cafestol and kahweol, found in coffee beans [48,49]. However, the exact mechanism through which cafestol and kahweol influence cholesterol levels is not well understood. In vitro studies have produced conflicting results across different cell lines [50,51,52]. Therefore, to maximize the cholesterol-lowering effectiveness of coffee, special extraction, processing, or proper brewing methods may be necessary.

## 4. Conclusions

In conclusion, this study highlights the promising potential of Arabica and Robusta coffee leaf extracts as natural inhibitors of pancreatic lipase and cholesterol absorption. Despite the similar nutritional profiles of the two extracts, their bioactive compounds, specifically CGA and caffeine, exhibit distinct inhibitory effects on lipase activity and cholesterol micellar solubility. Notably, the Arabica extracts showed a higher efficacy in lipase inhibition, while the Robusta extracts were more effective in reducing cholesterol uptake. The binding affinities of CGA and caffeine to the cholesterol-binding site of NPC1L1 suggest their role in impeding cholesterol absorption. These findings pave the way for the development of coffee leaf extracts as functional ingredients in dietary interventions aimed at managing obesity and hyperlipidemia, offering a natural and accessible approach to promoting cardiovascular health.

## Figures and Tables

**Table 1:** Nutritional values of coffee leaves’ extracts.

Leaf	%Moisture	%Dry Matter	%Fiber	%Ash	%Fat	%Protein	%NFE	Energy/100 g (Kcal)
Arabica	7.20 ± 0.85	99.28 ± 0.01	85.63 ± 0.25	5.59 ± 0.03	1.53 ± 0.04	17.73 ± 0.23 *	18.03 ± 0.74 *	425.70 ± 2.26
Robusta	7.38 ± 0.05	99.10 ± 0.06	85.64 ± 0.03	6.99 ± 0.17 *	1.22 ± 0.04	14.33 ± 0.15	16.15 ± 0.40	425.80 ± 4.38

Values are expressed as mean ± SEM (*n* = 3); * indicates a significant difference between groups.

**Table 2:** Quantitative HPLC analysis.

Leaf	Chlorogenic Acid (mg/g Extract)	RT (min)	Caffeine (mg/g Extract)	RT (min)
Arabica	2.91 ± 0.22	27.997	19.82 ± 0.75	27.989
Robusta	18.15 ± 0.91	27.970	14.90 ± 0.76	28.679

Values are expressed as mean ± SEM (*n* = 3).

**Table 3:** Determination of IC_50_ values of Arabica and Robusta coffee leaf extracts for the inhibition of bile acid binding and cholesterol micellar solubility at increasing inhibitor concentrations.

Substance	Bile Acid Binding (IC_50_) (µg/mL)			Micellar Solubility Inhibition (IC_50_) (µg/mL)
	TC	TD	GC	
Arabica coffee leaf extract	1804.00 ± 1.26	4905.00 ± 3.10	998.00 ± 1.07	2527.00 ± 1.09
Robusta coffee leaf extract	2215.00 ± 1.20	4760.00 ± 2.71	828.70 ± 1.08	2542.00 ± 1.18
Cholestyramine	1050.00 ± 1.08	642.40 ± 1.12	1039.00 ± 1.09	602.90 ± 1.07

Values are expressed as mean ± SEM (*n* = 3). Abbreviations: TC, taurocholic acid; GC, glycodeoxycholic acid; TD, taurodeoxycholic acid.

**Table 4:** Affinity for binding to the N-terminal domain of NPC1L1 and the molecular characteristics of ligands, chlorogenic acid, and caffeine.

No.	Protein	Ligand	Binding Energy (kcal/mol)	No of H-Bonds	Amino Acid Involved in Interaction	Intermolecular H-Bonding Distance (Å)
1	NPC1L1-NTD	Chlorogenic acid (CGA)	−6.1	4	MET50 LEU52 THR106 THR187	2.0 2.0 2.4 2.4
2	NPC1L1-NTD	Caffeine	−5.1	1	SER 53	3.0

## Data Availability

The original contributions presented in the study are included in the article, further inquiries can be directed to the corresponding author.
